# Characterization of the Heat Shock Transcription Factor Family in *Lycoris radiata* and Its Potential Roles in Response to Abiotic Stresses

**DOI:** 10.3390/plants13020271

**Published:** 2024-01-17

**Authors:** Ning Wang, Xiaochun Shu, Fengjiao Zhang, Guowei Song, Zhong Wang

**Affiliations:** 1 Institute of Botany, Jiangsu Province and Chinese Academy of Sciences (Nanjing Botanical Garden Mem. Sun Yat-Sen), Nanjing 210014, China; wangning813@njau.edu.cn (N.W.); sxc@cnbg.net (X.S.); fengjiao@cnbg.net (F.Z.); sgw12311@163.com (G.S.); 2 Jiangsu Key Laboratory for the Research and Utilization of Plant Resources, Jiangsu Provincial Platform for Conservation and Utilization of Agricultural Germplasm, Nanjing 210014, China

**Keywords:** *Lycoris radiata*, HSF transcription factors, expression patterns, hormone, abiotic stress, subcellular localization

## Abstract

Heat shock transcription factors (HSFs) are an essential plant-specific transcription factor family that regulates the developmental and growth stages of plants, their signal transduction, and their response to different abiotic and biotic stresses. The HSF gene family has been characterized and systematically observed in various species; however, research on its association with *Lycoris radiata* is limited. This study identified 22 *HSF* genes (*LrHSFs*) in the transcriptome-sequencing data of *L. radiata* and categorized them into three classes including HSFA, HSFB, and HSFC, comprising 10, 8, and 4 genes, respectively. This research comprises basic bioinformatics analyses, such as protein sequence length, molecular weight, and the identification of its conserved motifs. According to the subcellular localization assessment, most LrHSFs were present in the nucleus. Furthermore, the *LrHSF* gene expression in various tissues, flower developmental stages, two hormones stress, and under four different abiotic stresses were characterized. The data indicated that *LrHSF* genes, especially *LrHSF5*, were essentially involved in *L. radiata* development and its response to different abiotic and hormone stresses. The gene–gene interaction network analysis revealed the presence of synergistic effects between various *LrHSF* genes’ responses against abiotic stresses. In conclusion, these results provided crucial data for further functional analyses of *LrHSF* genes, which could help successful molecular breeding in *L. radiata*.

## 1. Introduction

Heat shock transcription factors (HSFs) are widely expressed proteins in various species, including fungi, animals, yeast, bacteria, and plants [1,2,3,4,5,6]. However, their number varies greatly between different organisms, such as sessile plants and animals. Vertebrates and drosophila have only three and one *HSF* genes, respectively, whereas *Arabidopsis thaliana*, rice (*Oryza sativa*), wheat (*Triticum aestivum*), potato (*Solanum tuberosum*), maize (*Zea mays*), kiwifruit (*Actinidia eriantha*), and rye (*Secale cereale* L.) contain 21, 25, 82, 27, 25, 41, and 31 *HSF* genes, respectively [7,8,9,10,11,12,13,14]. The HSF comprises a markedly conserved structure and functional domains, such as an N-terminal DNA-binding domain (DBD), a nuclear export signal (NES), an N-terminal adjacent bipartite oligomerization domain (OD), a nuclear localization signal domain (NLS), and a C-terminal transcriptional activation domain (CTAD) [14,15,16,17]. The most conserved among these are DBD and OD. The DBD is present at the N-terminus and has a helix-turn-helix hydrophobic structure, which recognizes and binds the target gene’s promoter heat shock element (HSE) motif [17,18,19]. The plant’s HSF family has also been categorized into three classes (HSFA, HSFB, and HSFC) on the basis of their basic amino acid (aa) sequences’ length between the DBD and OD regions and the number of aa residues inserted into the OD regions [7]. The OD comprises HR-A and HR-B, hydrophobic heptapeptide repeat regions, which modulate protein–protein interactions (PPI) during transcriptional activation and are also linked with nuclear import and export [20]. HSFs regulate gene expression to maintain cellular homeostasis and control the heat stress (HS) response as well as other environmental factors. During stressful conditions, *HSFs* activate transcription, and their accumulation serves as chaperones to fold damaged proteins and save new ones [21].

Furthermore, it has been demonstrated that HSF members are markedly associated with plant development and various stress responses, such as responses to freezing and salt, drought, and high-temperature tolerance [5,7,22,23,24,25,26]. In plants, *HSF* genes are the primary modulator of high-temperature stress [22]. Much research has been focused on elucidating the genetic role of HSF in plants during heat stress. Moreover, the HSFA members are essentially involved in HS responses in Arabidopsis [26], *Passiflora edulis* [27], tomato [28], *Zea mays* [29], wheat [30], and *Apium graveolens* [31]. Additionally, HSF regulates plant’s resistance to low temperatures, such as *AtHSFA6a*, *AtHSFA6b*, *AtHSFA9*, *AtHSFC1*, *BraHSF039*, *BraHSF043*, *OsHSFA3*, *OsHSFA4d*, *OsHSFA7*, *OsHSFA9*, *OsHSFC1*, and *OsHSFC2b* from *Arabidopsis*, cabbage, and rice [4,17,32,33,34]. Various *HSF* genes modulate plants’ adaptive response to drought [6,35,36]. It has been observed that drought and salt conditions markedly induced *AtHSFA6B*, an A6 subgroup member, and positively modulated *Arabidopsis* tolerance to abscisic acid (ABA)-mediated drought, salt, and HS [37]. Some *HSF* genes have been identified to affect plants’ resistance to drought negatively, for instance, *OsHsfB4b* in rice [38]. Like drought stress, HSF members’ expression varies during various salt treatment stages. It has been observed that when the leaves of strawberries are treated with NaCl, the level of *FvHsfA2a* was enhanced in the early stress stage, whereas that of *FvHsfA3a*, *FvHsfA5a*, and *FvHsfA9a* elevated during the late or middle stages [39]. Consistently, various HSF family members can positively or negatively regulate responses against salt stress. Moreover, levels of *AtHsfA2* and *AtHSFA7b*, *OsHsfA2e* and *OsHsfA7*, as well as *TaHsfA2d* elevate salt tolerance in *Arabidopsis*, rice, and wheat, respectively [40,41,42,43], whereas in maize, *ZmHsf08* negatively regulates drought and salt stresses [44]. *HsfA4* communicates with other *HsfAs*, including *HsfA9* and *HsfA5*, to elevate the plant’s resistance to drought, heat, excessive zinc, salt, and cadmium stresses by increasing antioxidant capacity [45,46,47,48,49,50,51]. Therefore, *HSFs* are crucial in various stress-linked pathways.

The *Lycoris radiata* (L’Her.) Herb is a perennial bulb plant that is a member of the Amaryllidaceae family and is found in Northeast Asia, including China, South Korea, and Japan [52]. It is markedly employed for preparing traditional medicine. Currently, >110 potent, structurally distinct Amaryllidaceae alkaloids have been identified or isolated for phytochemical and pharmacological research. Furthermore, it is primarily utilized for its perennial herbaceous flowers as they have good ornamental properties. It is barren- and drought-resistant, water-saving, and cold-tolerant. Additionally, *L. radiata* has significant tolerance against abiotic stress, including cold, drought, and soil impoverishment stresses. However, a comprehensive analysis of drought resistance in *Lycoris* has not been performed. Currently, sucrose degradation as well as Amaryllidaceae alkaloid and anthocyanin biosynthesis has been studied in *Lycoris* plants via transcriptome sequencing [52,53,54,55,56,57]. There are few studies on stress-resistant genes, and they are limited to the detection of gene expression, and the function of gene has not been verified in *Lycoris* Our previous transcriptome research identified 22 *LrHSF* genes in *L. radiata* [52] and analyzed their motif pattern as well as the phylogenetic association of *Arabidopsis* with *L. radiata*. The assessment of subcellular localization indicated that LrHSF proteins were primarily present in the nucleus. Furthermore, the expression *LrHSF* gene under stress and hormone treatment was assessed by qRT-PCR, which indicated different expressions in different tissues, validating the *LrHSF* genes’ biological role in *L. radiata*. In addition, the probable PPI of LrHSFs was also predicted. This research comprehensively assesses the *LrHSF* genes in *L. radiata* to provide novel data for screening crucial *LrHSF* genes during stress treatment and different development stages in *L. radiata* and to furnish a theoretical foundation for assessing the functions of HSF family genes in other species.

## 2. Results

### 2.1. Identification and Characterization of LrHSF Proteins in L. radiata

The prediction of HSF superfamily genes was vastly duplicated in *L. radiata*. BLASTP and HMMER 3.0 were employed to predict potential *L. radiata*’s LrHSF protein sequences in the transcriptome database with an E-value threshold of <1 × 10^−5^. Using NCBI, all the identified sequences were verified, and with the help of SMART, conserved complete HSF domains were determined. Altogether 22 LrHSF proteins (LrHSF1 to LrHSF22) were identified (Table S1a). The lengths of *LrHSF* genes’ CDS varied between 648 and 1407 bp. The amino acid length of LrHSF proteins ranged from 215 to 468 aa; their molecular weight was 24.92 to 51.35 kDa, whereas the isoelectric points were 4.77 to 8.95. The Plant-mPLoc, ProtComp 9.0, and WOLF PSORT were utilized to predict LrHSF proteins’ subcellular localization, which revealed that most of these proteins had nuclear localization, while LrHSF9 was located in the cytoplasm. The NCBI was utilized to predict the homologous alignment, which revealed that 8 LrHSF proteins had the highest sequence homology with HSF proteins in *Asparagus officinalis*. Moreover, LrHSF5 and LrHSF19 indicated the highest sequence similarity with the *Narcissus tazetta* subsp. Chinensis homologous protein. These results indicated that significant physical and chemical differences exist between LrHSF proteins.

### 2.2. Phylogenetic Analysis and Classification of LrHSF Proteins

**Figure 1:**
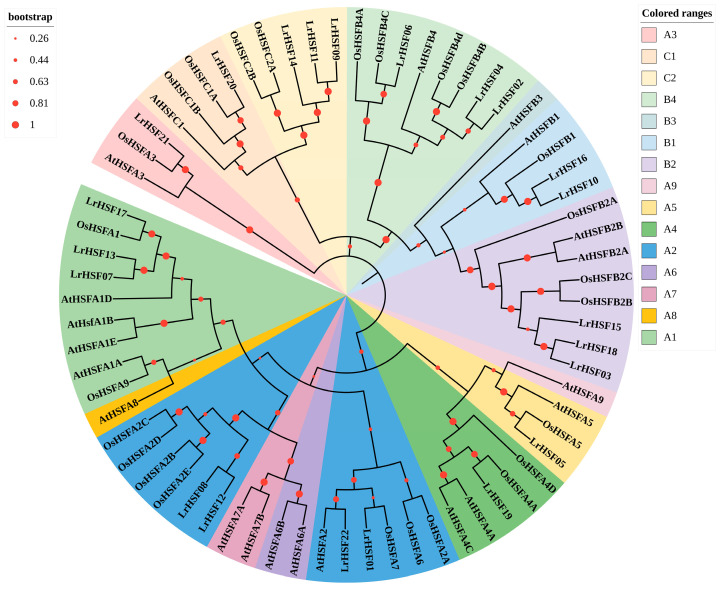
The phylogenetic assessment of HSF proteins in *L. radiata*. Using the neighbor-joining method, the phylogenetic tree was generated based on the HSF domain alignment. To verify the reliability, the numbers were computed via 1000 bootstrap replicates. The branches indicate replicate trees with >26%. The black font on a colored background depicts the subfamilies.

To investigate the evolutionary relationships and genetic characteristics between HSF proteins, a phylogenetic tree was established (Figure 1) with 22 *L. radiate*, 21 *Arabidopsis*, and 25 rice HSF proteins. According to the known *A. thaliana* and *O. sativa* HSF families, LrHSF could be categorized into 3 classes: HSFA, HSFB, and HSFC. As shown in Figure 1, LrHSF A was the largest class with a total of 10 members (LrHSF1, 5, 7, 8, 12, 13, 17, 19, 21, and 22) distributed across nine subclasses (A1–A9). LrHSF B comprised 5 subgroups (B1–B5) and 8 genes (*LrHSF2*, *3*, *4*, *6*, *10*, *15*, *16*, and *18*), whereas LrHSF C was the smallest class, comprising 4 genes (*LrHSF9*, *11*, *14*, and *20*). Interestingly, according to the phylogenetic tree, the HSF proteins of *L. radiata* were more closely clustered with those of *O. sativa*, suggesting that mono- and di-cotyledons HSF proteins have specific evolutionary variations.

### 2.3. Multiple Sequence Alignment and Conserved Motifs of LrHSF Proteins

**Figure 2:**
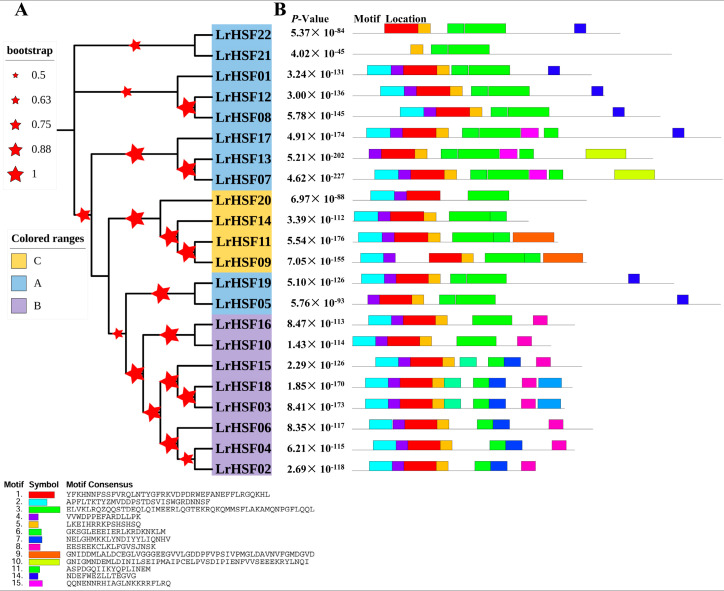
The phylogenetic relationships and conserved motifs analysis of LrHSF proteins. (**A**) Neighbor-joining LrHSFs phylogenetic tree (with bootstrap values calculated from 1000 replicates); (**B**) Distribution of the conserved motifs in LrHSF proteins. Various motifs are denoted by distinct colored boxes, where the length of each box corresponds to the length of the respective motif.

**Figure 3:**
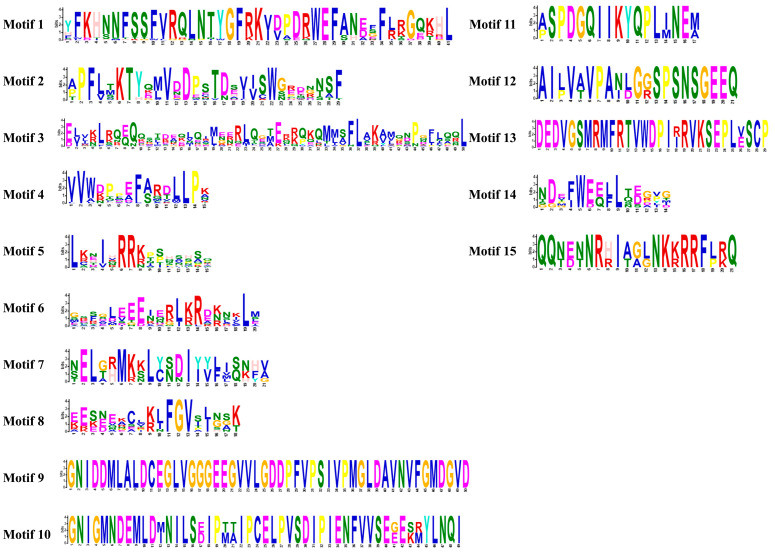
Motifs’ diversity and conservation and diversity in LrHSF proteins. Using MEME, 15 motifs in the HSF family were schematically represented.

**Figure 4:**
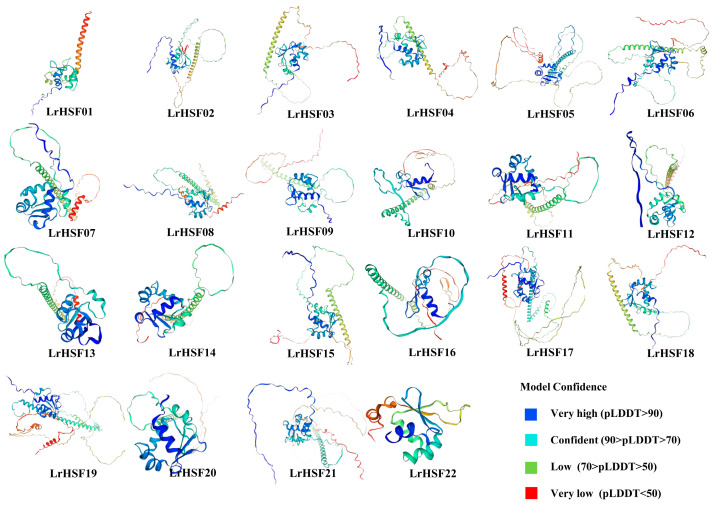
Protein structure of *L. radiata* HSFs. The confidence level of the prediction model is present at the bottom.

To further elucidate LrHSF proteins’ conservation and diversification, their conservative motifs were predicted via MEME Version 5.5.5, which indicated that LrHSF proteins comprised 15 different conserved motifs (Figure 2 and Figure 3). Motifs 1, 2, 4, and 5 were observed among the 22 LrHSF family members, whereas motifs 2 and 5 were identified in LrHSF22 only displayed, and LrHSF21 only displayed motif 5 (Figure 2). Furthermore, motifs 7 and 8 were exclusively identified in the HSFB, whereas 4, 10, 14, and 15 were only detected in HSFA. All HSFA members indicated motifs 1, 2, 3, 4, and 5 (Figure 3). Moreover, the HSFB members consistently indicated motifs 1, 2, 4, 5, and 8 and exclusively contained motifs 2 and 4. Each subgroup of LrHSF members mostly exhibited similar motifs; however, marked differences were observed between different subgroups. In the same subgroup, some proteins indicated similar motifs but unknown functions, suggesting a conserved protein structure in a specific subfamily of the LrHSF family. Motifs’ conserved pattern and phylogenetic assessment of the same group of proteins can be essentially utilized for basing protein classification. The protein secondary structure predicted that the DBD structural domain comprises four reverse parallel folds and three helix bundles. Additionally, the 3D structure of the *L. radiata* HSF protein was predicted, and the starting position of the one DBD structural domain was labeled (Figure 4).

### 2.4. Expression Patterns Analysis of LrHSF Genes in Different Tissues

**Figure 5:**
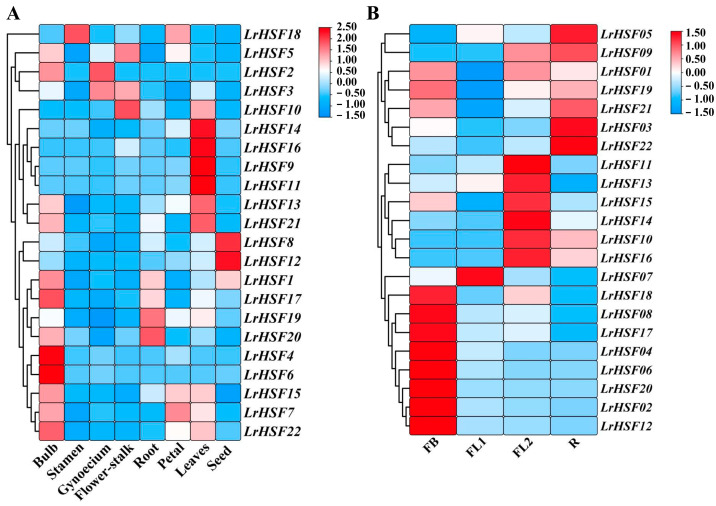
The expression of *LrHSF* genes in various *L. radiata* tissues. (**A**) Heatmap of expression profile with hierarchal clustering of *LrHSFs* in various *L. radiata* tissues and (**B**) at different flower developmental stages. The color intensity of each field depicts each gene’s relative expression. Red = higher values, and blue = lower values. FB: floral bud stage, FL1: partially opening flower stage, FL2: fully opened flower stage, R: senescent flower stage.

To evaluate LrHSF proteins’ biological role during various organs’/tissues’ developmental stages of *L. radiata*, the spatial specificity expression of 22 *LrHSF* genes in 8 *L. radiata* organs was assessed via qRT-PCR. As Figure 5A indicates, within each of the 8 tissues, some *LrHSF* genes exhibited differential expression, whereas in diverse tissues, similar expression patterns were observed, attributing the functional variation of these genes during plant development. For example, four *LrHSFs* (i.e., *LrHSF4*, *LrHSF9*, *LrHSF11*, and *LrHSF16*) had relatively increased expression in leaves. *LrHSF7* was specifically indicated in the petal; *LrHSF2* had elevated expression in the gynoecium, whereas *LrHSF4* and *LrHSF6* had relatively enhanced levels in the bulb. Additionally, *LrHSF18* was substantially expressed in stamen tissues. In particular, predominantly expression of *LrHSF19* and *LrHSF20* was observed in roots, of *LrHSF8* and *LrHSF12* in seeds, and of *LrHSF5* and *LrHSF10* in the flower stalk. The bulb and leaves indicated the least expression of *LrHSFs*, and some genes were specifically not expressed. In particular, it was observed that *LrHSF9* and *LrHSF11* had similar expression patterns, suggesting their functional similarity. These data suggested that *LrHSFs* might be essentially involved in the growth and development of *L. radiata*. Furthermore, the tissue-specific expression patterns of most *LrHSFs* were diverse, implying their diverse functions in different organs. 

**Figure 6:**
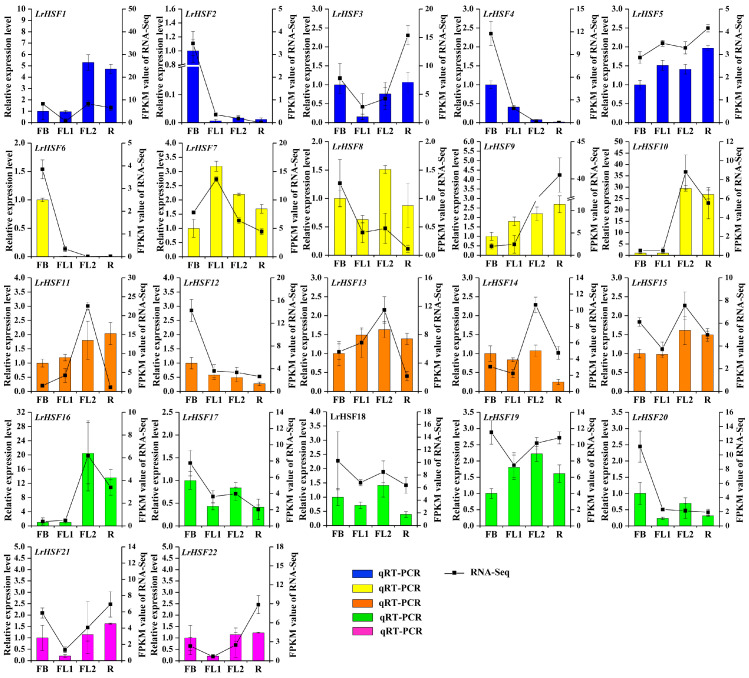
The expression of *LrHSF* genes at different flower developmental stages by qRT–PCR. FB: floral bud stage, FL1: partially opening flower stage, FL2: fully opened flower stage, R: senescent flower stage.

Based on the tissue-specificity of *LrHSFs*, its expression in the flowering developmental stages was assessed with the help of previous RNA-seq literature and the qRT-PCR technique (Figure 5B and Figure 6, Table S1b). During the *L. radiata* flowering development stage FB, FL1, FL2, and R indicated floral bud stage, partially opening flower stage, fully opened flower stage, and senescent flower stage, respectively (Figure 5B). During the FB stage, more than half of *LrHSF* genes indicated increased expression, which notably reduced at the FL1 stage. *LrHSF9* indicated a gradual increase in its expression during flower development, whereas the *LrHSF7* gene indicated increased expression during the FL1 stage, suggesting that it may have diverse activities. Furthermore, the expression of *LrHSF2*, *LrHSF4*, *LrHSF6*, *LrHSF8*, *LrHSF12*, *LrHSF17*, *LrHSF18*, and *LrHSF20* was also relatively increased during the early flower development stages. As shown in Figure 6, the expression levels of 22 *LrHSF* genes were normalized to the expression of reference gene *LrTIP41* by qRT–PCR analysis, and comparisons between the RNA-Seq data and qRT–PCR results were also conducted at different flower developmental stages of *L. radiata*, showing their good agreements (Figure 6).

### 2.5. Expression Analysis for LrHSFs Genes under Different Abiotic Stresses

**Figure 7:**
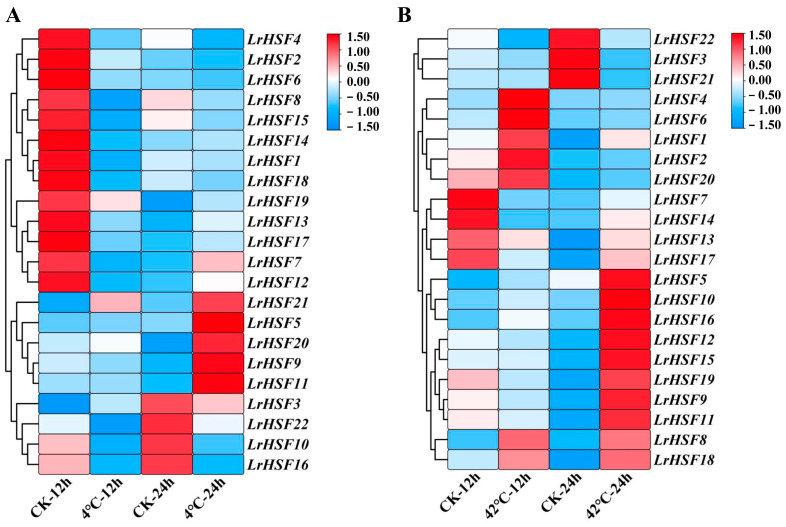
The expression of the *LrHSF* genes under cold and heat stresses in *L. radiata*. (**A**) Heatmap with hierarchical cluster assessment of differentially expressed *LrHSF* genes that were cold-responsive. (**B**) Heatmap of *LrHSF* gene expression in *L. radiata* during heat treatment. Leaf of seedling grown in distilled water under normal growth conditions for 12 h (CK-12 h) or 24 h (CK-24 h) was sampled as control. Red = higher values, and blue = lower values.

**Figure 8:**
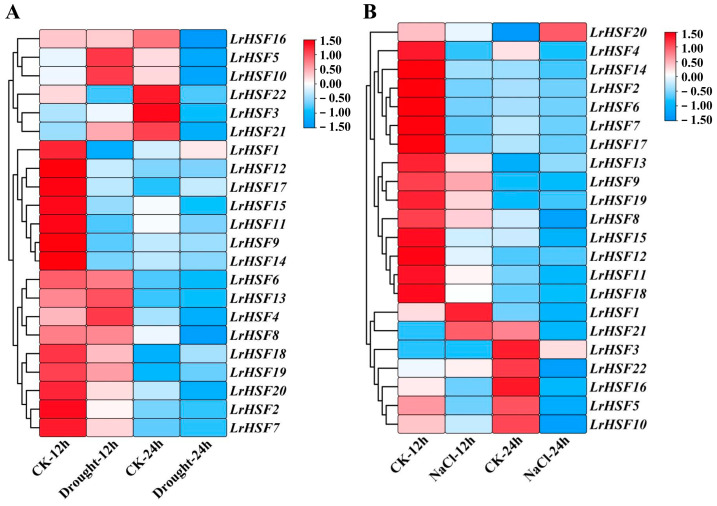
The expression pattern of *LrHSF* genes was tested during drought and salt stresses in *L. radiata*. (**A**) Heatmap of *LrHSF* gene expression during drought treatment. (**B**) Heatmap with hierarchical cluster assessment of differentially expressed *LrHSF* genes that were salt-responsive. Leaf of seedling grown in distilled water under normal growth conditions (22 °C for 8 h dark and 25 °C for 16 h light cycle) for 12 h (CK-12 h) or 24 h (CK-24 h) was set as the control. Red = higher values, and blue = lower values.

To investigate whether different abiotic stresses restricted the expression of *LrHSF* genes, the *LrHSF* genes’ expression patterns under cold, drought, heat, and salt stresses were analyzed with qRT-PCR (Figure 7 and Figure 8). The results showed that the functional genes in *L. radiata* during various stresses were slightly different than the control. Furthermore, some *LrHSF* genes exhibited marked up- and down-regulated expression under different stresses. Most *LrHSF* genes also indicated marked differences in leaves during the treatment period. For instance, cold stress induced most *LrHSF* genes, whereas during heat stress, most genes were expressed in leaves. Notably, *LrHSF2*, *LrHSF4*, *LrHSF6*, *LrHSF8*, *LrHSF10*, *LrHSF15*, *LrHSF16*, and *LrHSF21* indicated alternate expression patterns compared to heat and cold stress in leaves (Figure 7). The expression of *LrHSF5*, *LrHSF9*, *LrHSF11*, *LrHSF20*, and *LrHSF21* was markedly up-regulated under cold stress and mostly concentrated at 24 h treatment time (Figure 7A). During heat stress, *LrHSF5*, *LrHSF8*, *LrHSF9*, *LrHSF10*, *LrHSF11*, *LrHSF12*, *LrHSF15*, *LrHSF16*, *LrHSF18*, and *LrHSF19* were notably up-regulated at 24 h in leaves (Figure 7B). Moreover, most genes also indicated a markedly increased expression under salt and drought stresses (Figure 8). In *L. radiate*, *LrHSF5* and *LrHSF10* responded to drought, whereas *LrHSF1* and *LrHSF21* responded to salt stress at 12 h in leaves. Additionally, some *LrHSF* genes were notably up-regulated and down-regulated during different stressors, and most of these genes were substantially different in leaves with the treatment period. 

**Figure 9:**
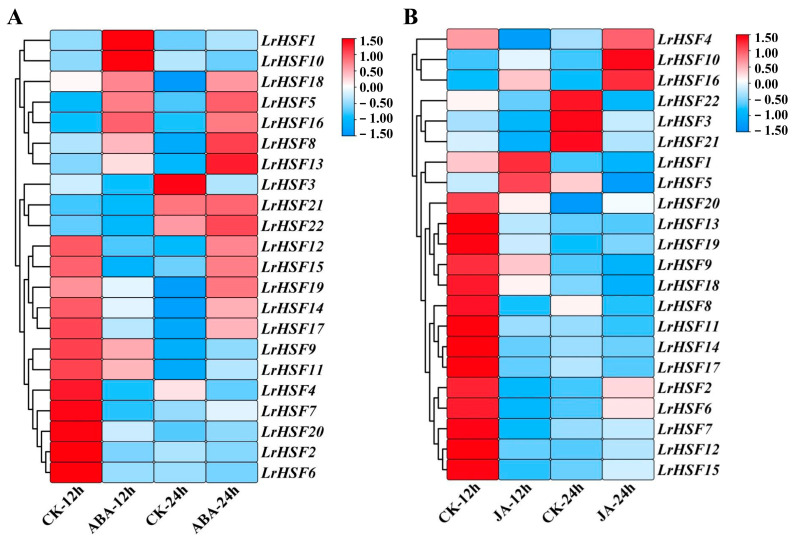
The expression of 22 *LrHSF* genes in seedling-stage leaves under ABA (**A**) and MeJA (**B**) treatments was assessed via qRT-PCR. Red = higher values, and blue = lower values.

Furthermore, the expression of *LrHSFs* in *L. radiata* leaves under ABA and MeJA treatment was assessed to further elucidate the genes’ functions, indicating differences in expression patterns after hormone treatments (Figure 9). For example, after ABA treatment, most genes were down-regulated, while *LrHSF5*, *LrHSF8*, *LrHSF13*, *LrHSF16*, and *LrHSF18* were up-regulated at 24 h in *L. radiata* leaves. Moreover, during MeJA treatment, *LrHSF1* and *LrHSF5* indicated the highest expression at 12 h, while *LrHSF1* and *LrHSF5* were down-regulated at 12 h and 24 h in *L. radiata* leaves. Additionally, the expression of *LrHSF10* and *LrHSF16* was increased during MeJA treatment.

### 2.6. LrHSF Proteins’ PPI Networks

**Figure 10:**
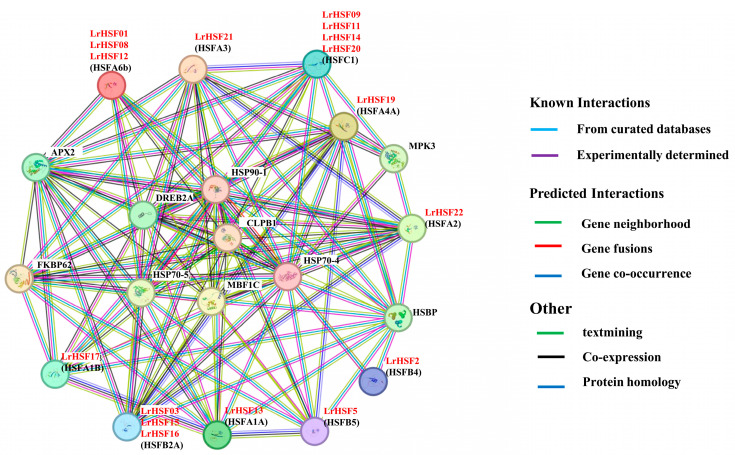
The STRING database predicted protein–protein interaction networks between LrHSFs. Different colors indicate distinct interactions. *Arabidopsis* HSF names are marked, whereas in the parentheses their *L. radiata* homologs are indicated.

The STRING database predicted various LrHSF proteins linked with each other on the basis of the orthologs of Arabidopsis, consistent with the literature suggesting that the binding activity of HSF proteins relies on the formation of homo- or hetero-dimers among HSF proteins (Figure 10, Tables S2 and S3). Several key interactions were then predicted. Using STRING 11.5 *e*-software, an HSF PPI network was established to identify PPIs. Altogether, 8 high-confidence interacting HSF family proteins were identified in the Arabidopsis. For instance, LrHSF22 is substantially homologous to HSFA2 of Arabidopsis, suggesting that it may potentially interact with plant defense proteins APX2 (ascorbate peroxidase 2), ClpB1 (Caseinolytic protease B1), DREB2A (dehydration-responsive element-binding 2A), HSP70 (heat shock protein 70), HSP90 (heat shock protein 90), and MPK3 (mitogen-activated protein kinase 3) more strongly. Similarly, the LrHSF03, LrHSF15, and LrHSF16 proteins were highly homologous to HSFB2A, the LrHSF12 and LrHSF17 proteins were highly homologous to HSFA1A and HSFA1B, LrHSF13 was to HSFA1A, and LrHSF21 was to HSFA3 of Arabidopsis. It is presumed that these proteins have a stronger interaction with the internal HSF family members. In this investigation, the HSF members indicated an expanded regulation network, suggesting that these genes might be crucial for sensing and responding to abiotic stresses. These results partially confirmed the hypothesized interaction networks and suggested their comparable roles in *L. radiata*.

### 2.7. Subcellular Localization of LrHSF Proteins

**Figure 11:**
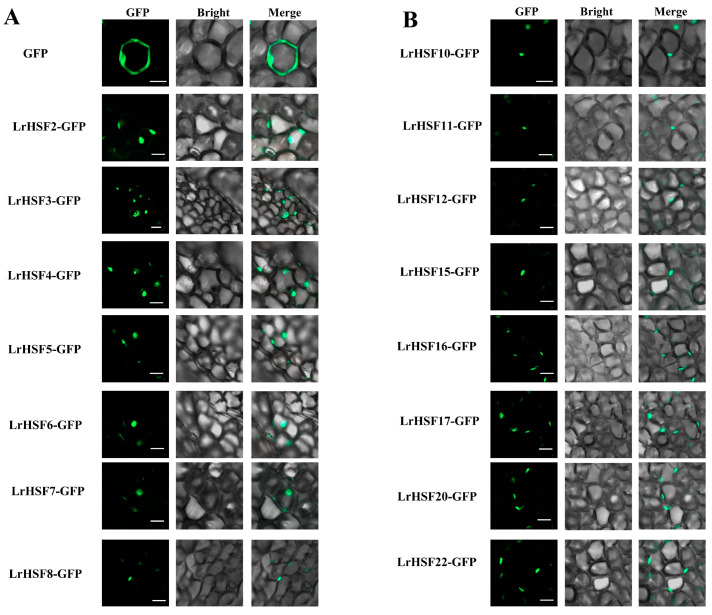
The *L. radiata* petal cells indicated transient expression of LrHSFs that had subcellular localization using GFP as a control. (**A**,**B**) The images were acquired via bright channel, green channel (GFP fluorescence), and their combination under a confocal microscope. Merged green fluorescence, bright-field, and green-bright fluorescence images. Scale bar = 10 μm.

The online software WolfPsort, ProtComp 9.0, and Plant-mPLoc predicted most LrHSF proteins were present in the nucleus, whereas LrHSF9 was located in the cytoplasmic (Table S1a). To validate the subcellular localization of certain LrHSF proteins, such as LrHSF2–8, 10–12, 15–17, 20, and 22, they were transiently transformed in *L. radiata* petal cells using 35S-GFP construct as a positive vector (Figure 11). LrHSF2–8, 10–12, 15–17, 20, and 22 fusion proteins were observed in the nucleus, thus validating that most LrHSFs are nucleoproteins.

## 3. Discussion

### 3.1. Characterization of the LrHSF Gene Family in L. radiata

Recent rapid advancements in metagenomics have identified and characterized HSF genes in multiple plants, such as *A. thaliana* [15], rice [8], potato [9], *Brachypodium distachyon* [58], *Triticum aestivum* [59], and *Zea mays* [60]. However, the LrHSF family remains to be elucidated. This research identified 22 *LrHSF* genes in *L. radiata* with HSF proteins comprising 215 to 468 amino acids (Table S1a). According to the conserved structural domains, *Arabidopsis* was categorized into three subgroups (Figure 1). Additionally, in *L. radiate*, all the HSF fragment duplication genes were from the same subfamily, suggesting that they may have similar functions. The literature suggests that the *HSFs* of class A were the primary modulatory factors during heat stress, while classes B and C members indicated no transcriptional activity [39,61]. Numerous researches validated that in the HSF gene family, *HSFA2* and *HSFA7* are the most heat-sensitive transcription factors [62]; they respond to heat stress by modulating plant hormones [63], protein synthesis [64], and ROS signaling pathways [65]. The phylogenetic tree predicted the biological role of unknown genes using known gene activities. A substantial *HSF* gene collinearity was observed between *L. radiata* and *Arabidopsis*, such as *LrHSF1*/*AtHSFA2*, *LrHSF8*/*AtHSFA2*, and *LrHSF12*/*AtHSFA2* (Figure 1; Supplementary Table S3). It has been observed that *AtHSFA2* modulates the ABA pathway to negatively control *A. thaliana* heat resistance [66], inferring that *LrHSF1*, *LrHSF8*, and *LrHSF12* might be linked with heat stress. Additionally, *LrHSF8* and *LrHSF12* corresponded to rice *HSF* genes (*OsHsfB2b*), which negatively modulated salt and drought tolerance [67]. The direct homologous gene of *LrHSF7*, *LrHSF13*, and *LrHSF17* is *OsHSFA1*, which promotes plants’ reproductive development and improves stress resistance [68]. Therefore, these *LrHSF* homologs may have similar functions under stress. Protein homology was found through the normal mode option for 3D modeling (Figure 4). The N-terminal of HSF [69] mainly consists of an α-helix structure. A study about chaperonins demonstrated that their α-helical structure facilitates the folding of a protein faster [69]. The results of the recent study are compatible with rice [8], sunflower [69], and jujuba [70].

### 3.2. Expression Patterns and Function Prediction Analysis of LrHSFs

The *HSF* genes are markedly linked with plants’ development and growth. Here, the expression of selected 22 *LrHSF* genes was assessed in various tissues and developmental stages. It was indicated that the *LrHSF* genes were markedly expressed in *L. radiata*. It has been reported that most HSF transcription factors regulate *Arabidopsis* development. For example, *AtHSFB2A* from subgroup B2 modulates its gametophyte development [68]. *LrHSF18* and *AtHSFB2A* were clustered in subgroup B2, and both were highly expressed in stamen (Figure 5), suggesting their similar functionality. Moreover, the overexpressing transgenic lines of *AtHSFB4* indicated a shorter root length phenotype than the wild type, suggesting its involvement in the negative root development modulation [71]. Furthermore, in subgroup B4, *AtHSFB2A* was also clustered with *LrHSF2*, *LrHSF4*, and *LrHSF6*, all of which showed reduced expression in roots (Figure 5 and Figure 6), suggesting that *LrHSF2*, *LrHSF4*, and *LrHSF6* might have similar biological functions in *L. radiata* roots. 

In plants, HSF proteins essentially regulate responses against various abiotic stresses, for instance, the response against drought, cold, salt, and high temperature [19]. This research found that most *LrHSF* genes were markedly up-regulated in leaves during cold and heat treatments, while some were up-regulated during drought and salt treatments (Figure 7 and Figure 8). This elaborates on the increased adaptability of *L. radiata* in alpine or arid regions. In Arabidopsis, most heat stress-response gene expressions are modulated by *AtHsfA1* [61,72,73]. It has been observed that *AtHsfA1b* and *AtHsfA1d* increase heat and drought tolerance, respectively [74,75], whereas *AtHsfA2* and *AtHsfA3* modulate heat tolerance, and *AtHsfA2* also elevates anoxia tolerance [76,77,78,79,80]. Furthermore, in *L. radiate*, 10 *LrHSF* genes (*LrHSF5*, *LrHSF8*, *LrHSF9*, *LrHSF10*, *LrHSF11*, *LrHSF12*, *LrHSF15*, *LrHSF16*, *LrHSF18*, and *LrHSF19*) were observed to be markedly up-regulated under heat tolerance (Figure 7B). Some studies indicated that in plants, *HSF* genes also react against cold stress. For example, 6 *PvHsf* genes in common bean and 5 *VviHsf* genes in wild Chinese grapevine respond to cold stress [81,82]. This investigation identified 5 *LrHSF* genes (*LrHSF5*, *LrHSF9*, *LrHSF11*, *LrHSF20*, and *LrHSF21*) that responded to cold stress in *L. radiata* (Figure 7A). In *Tamarix hispida*, salt tolerance was positively modulated by *ThHSFA1*, which directly stimulated *ThWRKY4* [83]. In rice, *OsHsfB2b* has been observed to negatively control salt and drought tolerances [66]. An investigation on *HSF* genes in carrots identified that 33 of these genes were down-regulated during drought stress, whereas during salt stress, three genes were up-regulated, suggesting that these genes might be associated with salt and drought stresses [17]. Here, 18 and 22 *LrHSF* genes were identified to respond to salt and drought stresses, respectively, whereas 18 genes responded to both these stresses (Figure 8). Additionally, *LrHSF5* expression was observed under all stresses, validating it as a potential candidate gene for improving crop breeding. Altogether, these data suggest that in plants *HSF* genes crucially modulate responses to various abiotic stresses.

### 3.3. PPI Network Prediction and Validation of LrHSFs

Numerous researches have revealed that HSF proteins play an important function for replaying to respond abiotic stress [31,84]. For example, HSFA1A (homolog of LrHSF13) and HSFA1B (homolog of LrHSF17) directly induce the expression of heat-stress-responsive transcription factors DREB2A, HSFA2 (homolog of LrHSF22), HSFA7s, and MBF1, further activating HSFA3 (homolog of LrHSF21) expression to maintain the heat stress response (Figure 10) [15,85]. *HSFA1* also negatively regulated the nuclear localizations and activities by *HSP70* and *HSP90*, whereas heat stress induces the accumulation of unfolded proteins that interact competitively with *HSP70* and *HSP90*, thereby causing *HSFA1s* to be released from the *HSP70* and *HSP90* complex and become active [86]. ClpB1 interacted with Hsp70, Hsps, and proteasomes during heat stress [87]. The survival tare of AtClpB1 mutant *hot1-3* is lower compared to wild type plants under heat tolerance [88]. MBF1, HSP70, HSP90, and DREB2A proteins were associated with abiotic stress responses, and ClpB1 protein is a critical component in governing tolerance against heat tolerance [88,89,90]. Furthermore, LrHSF13 (HSFA1A homologs) and LrHSF17 (HSFA1B homologs) were observed to be markedly up-regulated under heat tolerance at 24h in *L. radiata*. *LrHSF13* and *LrHSF17* might have a similar function in heat stress response. *HSFA2* is a regulator of multiple environmental stress responses required for stress acclimation, highly induced by *HSFA1s* following heat exposure, and strongly reduced the expression of *HSP18*, *HSP21*, *HSP22*, *HSA32* (heat-stress-associated 32 KD protein), and *APX2* [80,91,92]. APX2 proteins regulated plant oxidative damage and induced the expression of the ROS-scavenging-related gene for response against abiotic stress in Arabidopsis [93]. *LrHSF22* may have the similar functions with *HSFA2* and play conserved roles in the response to heat stress in plants. Plant HSF TFs are downstream components of the signal transduction pathway and maintain regulatory roles for stress-related gene expression [94]. It was hypothesized that nearly all LrHSFs localize to the nucleus so that they may perform transcriptional functions (Figure 11). Additional research is necessary to demonstrate the comprehensive interaction network of the *LrHSF* TFs during *L. radiata* growth and development.

## 4. Materials and Methods

### 4.1. Plant Materials and Plant Treatments

The *Lycoris radiata* (L’Her.) Herb was cultivated at the Institute of Botany, Jiangsu Province and the Chinese Academy of Sciences, Nanjing, China. The similar diameter size (2.8–3.2 cm) seedlings were transferred into plastic pots containing soil and vermiculite (*v*/*v*, 1:1) and incubated in a plant growth chamber with 120 μmol m^−2^ s^−1^ irradiation at 22 °C for an 8 h dark and 25 °C for a 16 h light cycle. The expression pattern of 22 *LrHSF* genes under different stresses was determined. Briefly, *L. radiata* seedlings were subjected to different abiotic stress treatments, including drought (20% PEG 6000), high-temperature (42 °C), salt (200 mM NaCl), and low-temperature (4 °C) stresses. For high-temperature and low-temperature stress, *L. radiata* seedlings were transferred into a plant light incubator set as 42 °C and 4 °C, respectively. The control seedlings incubated in the distilled water were maintained in the plant growth chamber under normal growth conditions. For hormonal treatments, a solution comprising abscisic acid (ABA, 0.1 mM) and methyl jasmonate (MeJA, 0.1 mM) was sprayed on the seedlings while the control was sprayed with distilled water. Post 12 and 24 h of treatment, all the treated leaves were sampled for RNA isolation. All the experiments were repeated thrice. Furthermore, using the flower stalks, petals, seeds, leaves, stamens, roots, gynoeciums, and bulbs, the tissue-specific transcription data of 22 *LrHSF* genes of these plants were analyzed. Before storage at −80 °C, all the samples were snapping frozen in liquid nitrogen.

### 4.2. Identification and Sequence Analysis of LrHsf genes in L. radiata

To screen the potential *LrHSF*, the *L. radiata* transcriptome dataset comprising 87,584 unigenes associated with the four flower development stages was used [52]. AtHSF proteins were imported from TAIR (*Arabidopsis* Information Resource database, https://www.arabidopsis.org/) (accessed on 7 June 2023) to elucidate database-acquired *L. radiata* transcripts’ sequence homology via the basic local alignment (BLASTn). Furthermore, the PFAM protein family database (http://pfam.sanger.ac.uk) (accessed on 9 June 2023) was employed to generate a Hidden Markov Model (HMM) comprising HSF domains (PF0047). The HMM model cutoff value of 0.01 was set in HMMER 3.0 to compare LrHSF protein sequences of *L. radiata* (http://plants.ensembl.org/hmmer/index.html) (accessed on 10 June 2023). Subsequently, the predicted *LrHSF* transcription factors’ HSF domain was verified via the NCBI Batch CD-Search Tool (https://www.ncbi.nlm.nih.gov/Structure/bwrpsb/bwrpsb.cgi) (accessed on 10 June 2023) under default settings. It was suggested that this characteristic will have an increased confidence relation with the conserved domain. For further assessment, the sequences that were identified as specific hits were selected (Table S1). Moreover, the databases SMART and PFAM (http://smart.embl-heidelberg.de/) (accessed on 11 June 2023) were employed to verify the HSF domain in each selected protein sequence. Lastly, with the help of the ExPASy (https://web.expasy.org/protparam) (accessed on 11 June 2023), the molecular sizes (MW), protein instability index, isoelectric points (PI), and the complete amino acid sequences were determined.

### 4.3. LrHSF Proteins’ Phylogenetic Tree and Motif Analyses

The neighbor-joining (NJ) protocol was utilized for establishing a phylogenetic tree of HSFs from *A. thaliana*, *O. sativa*, and *L. radiata*, using MEGA7.0 (https://www.megasoftware.net/) (accessed on 2 August 2023). Based on the phylogenetic association with OsHSF and AtHSF proteins, the LrHSF proteins were classified. The Multiple EM for Motif Elicitation (MEME, v 5.1.1), an e-tool, was employed to screen the conserved motifs (number and width of 14–50 aa for each gene) of LrHSF proteins (https://meme-suite.org/meme/tools/meme) (accessed on 5 August 2023). Moreover, the motifs were also searched using the SMART program (http://smart.embl.de/) (accessed on 11 August 2023). To predict the protein secondary structure, the Jalview output was submitted to SOPMA (https://npsa-prabi.ibcp.fr/cgi-bin/npsa_automat.pl?page=npsa_sopma.html) (accessed on 15 August 2023), using default parameters. Using the online program AlphaFold, available at https://alphafold.ebi.ac.uk/ (accessed on 30 August 2023), the 3-D structure of the *LrHSF* genes was predicted, as shown in Figure 4.

### 4.4. Total RNA Extraction, cDNA Reverse Transcription, and qRT-PCR Analysis

For whole RNA isolation, the RNA prep Pure Plant Kit (BC508, Huayueyang, Beijing, China) was employed per the kit’s guide. cDNA was generated via the PrimeScript™ II 1st Strand cDNA Synthesis Kit (TaKaRa Bio, Dalian, China), which was then used for relative gene expression levels assessment via qRT-PCR utilizing SYBR^®^ Premix Ex Taq™ II (Takara Bio, Dalian, China) on a Bio-Rad iQ5 Real-Time PCR System (Bio-Rad, Hercules, CA, USA). A prepared reaction was 15 μL and comprised ddH_2_O (5.9 μL) of 20 μM reverse and forward primers (0.6 μL, respectively), cDNA (1 μL), and 2×TransStart^®^ Top Green qPCR SuperMix (7.5 μL). The RT-qPCR protocol included the following: the PCR reaction conditions were at 95 °C for 5 min; denaturation 5 s at 95 °C; 60 °C for 30 s; 40 cycles. To normalize relative levels of target gene expression via the 2^−ΔΔCt^ method [95], the *LrTIP41* gene [52] was chosen to serve as the reference gene according to a previous study on *L. aurea* [96]. Supplementary Table S4 enlists the primer sequences used.

### 4.5. Gene Cloning and Expression Vector Construction

On the basis of the RNA-seq database acquired, the unigenes’ putative ORFs, *LrHSFs* were cloned. For ORF sequence amplification, primers were prepared using Tks GflexTM DNA Polymerase (Takara, Dalian, China) from *L. radiata* petal cDNA (Table S4). Reaction conditions were: 5 min of 95 °C, 40 cycles for 30 s at 95 °C, 30 s at 58 °C, 2 min at 72 °C, with extension at 72 °C for 10 min. The obtained PCR products were then cloned into pTOPO001 simple vectors (Genesand, Beijing, China). Thereafter, for amplification, the T-vectors were transfected into TOP10 competent cells (Genesand, Beijing, China). Using the One Step Cloning Kit (Genesand, Beijing, China), the *LrHSFs* overexpression vectors were established, and their ORFs were linked into a pBIN-MCS-GFP4 plant transformation vector. Lastly, *Agrobacterium tumefaciens* EHA105 competent cells were transfected with the 35S: *LrHSFs* recombinant vectors.

### 4.6. LrHSF Proteins’ Subcellular Localization Analysis

The subcellular localization of LrHSF proteins was predicted with the help of ProtComp 9.0 (http://linux1.softberry.com), WolfPsort (https://wolfpsort.hgc.jp), and Plant-mPLoc (http://www.csbio.sjtu.edu.cn/bioinf/plant-multi/) (all accessed on 11 August 2023). The pBinGFP4 plant expression vector was transfected with each *LrHSF* gene’s coding region. Then, *A. tumefaciens* strain EHA105 bacteria was transformed using this vector, which was then cultivated and harvested. The harvested plant was resuspended in an invasive solution (MES (10 mM), MgCl2 (10 mM), and Acetosyringone (0.2 mM)), with the final 0.6 OD_600_ value. For infiltration, *L. radiata* petals were utilized, which were then grown in the dark at 22 °C and transferred to standard conditions (22 °C /8 h dark and 25 °C /16 h light cycle) for 3 days. GFP signals were analyzed in *L. radiata* petal epidermal cells via a confocal microscope (Zeiss LSM900, Jena, Germany).

### 4.7. PPI Network Prediction of LrHSF Proteins

The potential PPI networks were predicted by the STRING.410 database (https://cn.string-db.org) (accessed on 11 August 2023) and on the basis of *A. thaliana* homologous proteins. Then, selecting *A. thaliana* as the comparative organism, sequences of 22 LrHSF proteins was submitted to the server (Table S2). The interaction network of *LrHSF* genes was established after blasting with the highest bitscore.

### 4.8. Statistical Analysis

All analyses were repeated thrice, and the data acquired were presented as mean ± SD. For data analysis, a Student t-test was carried out. * *p* < 0.05 and ** *p* < 0.01 were deemed significant.

## 5. Conclusions

In summary, identification and systematic analysis of *HSF* genes in *L. radiata* showed that the 22 *LrHSF* genes were classified into three subfamilies. A comprehensive assessment of conserved motifs of these 22 genes demonstrated similar motifs in the same, suggesting that they might have functional similarities. Furthermore, a preliminary structural analysis of *LrHSF* genes further detailed their expression pattern and indicated that *LrHSF* genes are crucial for *L. radiata* growth and development and respond to the hormonal and abiotic stresses during its development.

## Data Availability

All data are displayed in the manuscript.

## Supplementary Materials

The following supporting information can be downloaded at https://www.mdpi.com/article/10.3390/plants13020271/s1, Table S1. Characteristics of *LrHSF* genes identified from *L. radiata* transcriptome; Table S2. The full-length amino acids sequence of LrHSF proteins; Table S3. Summary information for LrHSF proteins in the STRING database; Table S4. Primers used in this study.
